# Supplementary material for: Anti-Allergic Diarrhea Effect of Diosgenin Occurs via Improving Gut Dysbiosis in a Murine Model of Food Allergy
Source: Molecules. 2021 Apr 23;26(9):2471. doi: 10.3390/molecules26092471 (PMC8122900; doi:10.3390/molecules26092471)
Supplement: Supplementary file 1 [file molecules-26-02471-s001.zip › molecules-1108571-supplementary.pdf]

molecules

Supplementary Item

Anti-Allergic Diarrhea Effect of Diosgenin Occurs via Improving Gut Dysbiosis in a Murine Model of Food Allergy

Chung-Hsiung Huang [1,*], Chorng-Liang Pan [1,2], Guo-Jane Tsai [1,2], Chun-Ju Chang [1,2], Wei-Chung Tsai [1] and Shueh-Yu Lu

[1] Department of Food Science, National Taiwan Ocean University, Keelung 20224, Taiwan; b0037@mail.ntou.edu.tw (C.-L.P.); b0090@mail.ntou.edu.tw (G.-J.T.); chunju@mail.ntou.edu.tw (C.-J.C.); 10932036@mail.ntou.edu.tw (W.-C.T.); 0043a029@email.ntou.edu.tw (S.-Y.L.)

[2] Center of Excellence for the Oceans, National Taiwan Ocean University, Keelung, Taiwan

* Correspondence: huangch@mail.ntou.edu.tw

Table 1. Individual score of diarrhea severity at each time of allergen challenge.

		Times of challenge					
Group		1st	2nd	3rd	4th	5th	6th
NA	NA1	0	0	0	0	0	0
		0	0	0	0	0	0
	NA2	0	0	0	0	0	0
		0	0	0	0	0	0
	NA3	0	0	0	0	0	0
		0	0	0	0	0	0
	NA4	0	0	0	0	0	0
		0	0	0	0	0	0
OVA	OVA1	0	1	1	1	2	3
		0	1	1	2	3	3
	OVA2	1	2	3	3	3	3
		1	2	2	3	3	3
	OVA3	1	1	2	2	3	3
		1	1	2	3	3	3
	OVA4	1	2	3	3	3	3
		1	2	3	3	3	3
DIO	OVADIO1	0	0	1	1	2	2
		0	0	1	2	2	2
	OVADIO2	0	0	0	0	0	0
		0	0	0	0	0	0
	OVADIO3	0	0	2	1	2	2
		0	0	1	1	2	2
	OVADIO4	0	0	0	0	1	1
		0	0	0	1	1	1

Mean score indicates the average of severity score from two mice in the same group with almost the same severity of diarrhea through the experiment. Subsequently, fresh fecal samples from the two mice were harvested and pooled together for NGS analysis and bacterial culture. Therefore, there are 4 fecal samples per group, and the samples are labeled as 1, 2, 3 and 4. In other words, the fecal samples of OVADIO group were named as OVADIO1, OVADIO2, OVADIO3 and OVADIO4.
